# Risk Factors and Biomarkers for Pulmonary Toxicities Associated with Immune Checkpoint Inhibitors

**DOI:** 10.3390/medicina61071258

**Published:** 2025-07-11

**Authors:** Efraim Guzel, Ismail Hanta, Oya Baydar Toprak, Okan Gurbuz, Burak Mete, Ertugrul Bayram

**Affiliations:** 1 Department of Chest Diseases, Faculty of Medicine, Cukurova University, 01330 Adana, Turkey; ismailhnt@gmail.com (I.H.); oyabaydarr@yahoo.com.tr (O.B.T.); okangrbz.96@gmail.com (O.G.); 2 Department of Public Health, Faculty of Medicine, Cukurova University, 01330 Adana, Turkey; burakmete2008@gmail.com; 3 Department of Medical Oncology, Faculty of Medicine, Cukurova University, 01330 Adana, Turkey; ertugrulbayram84@gmail.com

**Keywords:** immune checkpoint inhibitor, immunotherapy, pulmonary toxicity, organizing pneumonia, interstitial pneumonia, hypersensitivity pneumonitis

## Abstract

*Background and Objectives*: Immune checkpoint inhibitors (ICIs) have emerged as groundbreaking agents in cancer therapy; however, their immune-related adverse effects, especially pulmonary toxicity, significantly limit their use. This study aimed to determine the incidence and risk factors associated with ICI-induced pulmonary toxicity. *Materials and Methods*: We conducted a prospective observational study involving 126 patients aged ≥18 years with malignancies treated with ICIs between April 2022 and April 2024. Patients were followed every six months over a two-year period. Clinical, laboratory, and radiological data were collected to assess pulmonary toxicity. *Results*: The mean age of our patients was 62.93 ± 12.94 years, and 81% were male. The ICI-related pulmonary toxicity rate was 16.7%, and the all-cause mortality rate was 68.3%. In the analysis, the conditions associated with pulmonary toxicity were the type of malignancy, the presence of lung cancer, COPD, long-term ICI use, dyspnea, cough and sputum, the pre-ICI lung nodule mass, and high blood monocyte levels. Our regression analysis results for the determination of risk factors showed a 7.70-fold increase in the presence of cough symptoms, a 4.57-fold increase in the presence of COPD, a 0.998-fold increase for every 1 unit decrease in lymphocyte count, and an 11.75-fold increase in risk for a monocyte count of 130 or less. *Conclusions*: Our study’s findings suggest that patients with identifiable risk factors for pulmonary toxicity should undergo closer monitoring and early diagnostic evaluation during ICI therapy to reduce morbidity and mortality.

## 1. Introduction

Immunotherapy has revolutionized cancer treatment with the approval of ipilimumab in 2011, paving the way for the development of several immune checkpoint inhibitors (ICIs) for malignancy [1]. Critical for immune tolerance and response modulation, ICIs regulate immune cell activation and enhance immune responses to tumor antigens by disrupting inhibitory signals in the tumor microenvironment [1,2]. The first ICI, ipilimumab, targets cytotoxic T lymphocyte-associated antigen 4 (CTLA-4); later ICIs such as nivolumab, pembrolizumab, and cemiplimab target programmed death-1 (PD-1); and durvalumab, avelumab, and atezolizumab target programmed death-ligand-1 (PDL-1) [3]. These drugs, used as monotherapy or in combination, have shown remarkable efficacy in melanoma, lung cancer, urothelial cancer, and many other solid organ tumors, and their indications have continued to evolve. However, ICI therapy often triggers immune-related adverse events (irAEs), affecting almost all organs [4]. Specifically, adverse events in the melanoma occurred in 86% of patients treated with nivolumab, 86% of patients treated with ipilimumab, and 96% of those receiving combination therapy [5].

Common irAEs include cutaneous and gastrointestinal (GI) toxicity, hepatotoxicity, renal toxicity, endocrine toxicity, hematotoxicity, joint toxicity, neurotoxicity, cardiotoxicity, and pulmonary toxicity [3]. Some irAEs, particularly those causing colitis, are rare but can be fatal in the early stages of treatment with anti-CTLA-4 antibodies [6]. The diagnosis and management of irAEs depend on conventional testing and require the exclusion of non-ICI factors [7]. Pulmonary toxicities, which constitute a significant group of irAEs, usually present as pneumonitis, interstitial lung disease, pleural effusions, pulmonary sarcoidosis, and sarcoid-like granulomatous reactions [8]. Pneumonia may present as organizing pneumonia (OP) or cryptogenic organizing pneumonia (COP), nonspecific interstitial pneumonia (NSIP), hypersensitivity pneumonitis (HP), or usual interstitial pneumonia (UIP)/pulmonary fibrosis (PF) on imaging studies [8].

Given the increasing use of ICIs and the potential severity of pulmonary irAEs, early identification of high-risk patients is essential. Reliable risk factors and predictive biomarkers are urgently needed to facilitate timely diagnosis and intervention. In this study, we aim to identify clinical and laboratory predictors of ICI-induced pulmonary toxicity and assess their potential utility in clinical practice.

### 1.1. Existing Knowledge

Immune checkpoint inhibitors (ICIs), which have become increasingly prevalent in recent years, are now utilized across nearly all stages of cancer treatment. Despite their efficacy, ICIs are associated with a range of adverse effects, among which pulmonary toxicities are among the most severe and life-threatening. Several predisposing factors, including chronic obstructive pulmonary disease (COPD), smoking history, combination immunotherapy regimens, and decreased lymphocyte counts, have been identified as contributing to the development of pulmonary toxicity.

### 1.2. Novel Findings

Although the risk factors identified in our study largely align with those reported in prior research, our findings introduce novel diagnostic insights by identifying threshold values for key biomarkers such as the lymphocyte count, monocyte count, and C-reactive-protein-to-lymphocyte ratio (CLR), which may enhance early detection and risk stratification of ICI-induced pulmonary toxicity.

## 2. Materials and Methods

### 2.1. Study Design

This was a single-center, prospective observational study, conducted between April 2022 and April 2024 in the Oncology and Pulmonology outpatient clinics of Cukurova University Faculty of Medicine, Balcalı Hospital, Adana, Turkey. The study included consecutively enrolled patients aged ≥18 years with solid or hematological malignancies receiving ICIs. Written informed consent was obtained from all participants or their legal representatives prior to enrollment. The study was approved by the Cukurova University Non-Interventional Clinical Research Ethics Committee (approval number: 59/121, date: 8 April 2022) and was conducted in accordance with the Declaration of Helsinki and institutional ethical guidelines.

### 2.2. Participants

(a) Inclusion criteria were age older than 18 years, having a solid organ or hematologic malignancy, and the use of immune checkpoint inhibitors in monotherapy or in combination with chemotherapy.

(b) Exclusion criteria were refusal to participate in the study, being younger than 18 years of age, incomplete tests, the use of drugs with side effects that may cause radiological findings of interstitial lung diseases (OP, NSIP, HP), and the presence of suspected or established radiological findings of interstitial lung disease prior to ICI use.

### 2.3. Variables

Data collected included age, sex, height, weight, body mass index (BMI), smoking status, comorbidities, symptoms, radiological findings (chest X-ray and computed tomography), vital signs (temperature, heart rate, respiratory rate, blood pressure), oxygen saturation, cancer type and stage, treatment protocols (chemotherapy and immunotherapy drugs), and duration of ICI use. Laboratory parameters obtained prior to immunotherapy initiation were recorded. All data were collected via structured questionnaires and hospital electronic medical records.

### 2.4. Data Source/Measurement

Sociodemographic, clinical, laboratory, and radiological data were collected from patient interviews and the hospital information system. Pulmonary function tests (PFTs) were interpreted according to joint American Thoracic Society (ATS) and European Respiratory Society (ERS) guidelines. Obstructive patterns were defined by FEV_1_/FVC < 70%; restrictive patterns were defined by FVC < 80% with a normal FEV_1_/FVC ratio; and normal function was defined by FEV_1_/FVC ≥ 70% and FVC ≥ 80%. Comorbidities were categorized using the Charlson Comorbidity Index (CCI): low risk (0 points), moderate risk (1–2), high risk (3–4), and very high risk (≥5) [9].

### 2.5. Monitoring and Assessment

Patients were followed at three intervals: 6, 12, and 24 months after ICI initiation. Patients with radiological abnormalities due to infection or malignancy, or those receiving known pulmonary toxic medications, were excluded from the analysis. In the differential diagnosis of pulmonary toxicity associated with ICI, the most commonly confused conditions are those involving the lung parenchyma and interstitium, such as progression of lung malignancy, pulmonary infections, and pulmonary involvement of rheumatologic diseases. Radiologically, ground glass densities and consolidation findings, which are most commonly encountered, can occur in many conditions, especially infections, making the diagnosis of ICI difficult in this group of patients. The confusion in this patient group was resolved by taking into account clinical, laboratory, and radiologic features together. Diagnosis of pulmonary toxicity and identification of pathological images were assessed by expert radiologists at a tertiary academic center using thorax CT or high-resolution CT. Basically, 3 disorders were emphasized, and classifications were made under these headings. Peripherally located consolidations with ground glass or air bronchograms, diffuse bilateral infiltration, solitary focal mass, or diffuse nodular lesions suggestive of metastasis were defined as OP or COP. Radiological findings in cases with fine reticular infiltrates, patchy consolidation, and ground-glass opacities in the middle and lower lobes of the lungs and subpleural regions, but without honeycomb and traction bronchiectasis, were interpreted as NSIP or idiopathic interstitial pneumonia (IIP). Abnormalities less than 5 mm in size, with indistinct borders, numerous small nodules, patchy or diffuse ground glass opacities mimicking pulmonary edema, occasional fine reticulation, and, rarely, consolidation were defined as HP. However, the “three-density” finding, in which ground glass, normal density, and air trapping were observed together, was considered the most specific finding for HP [10]. Regardless of whether these interstitial patterns were characterized by radiologically local or diffuse involvement or clinically early or advanced lung involvement, all findings were associated with pulmonary toxicity. Clinical and laboratory findings were prioritized in differentiating the present radiological patterns from conditions that often cause similar findings, such as pneumonia or lymphangitic spread due to malignancy, and the diagnosis was supported by fiberoptic bronchoscopy when necessary. Lung parenchymal biopsy could not be performed.

### 2.6. Statistical Analysis

SPSS 22 program and Jamovi 2.6.17 version was used in the analysis of the data. The Kolmogorov–Smirnov test was used as normal distribution test. *t*-test, Mann–Whitney U test, Chi-square test, and binary logistic regression analysis were used in the analysis. Two logistic regression models were created. The Forward LR method was used in both models. In the first model, the effect of clinical risk factors/predictors on pulmonary toxicity prediction was examined. In the second model, the roles of inflammatory markers in pulmonary toxicity prediction were examined using a two-stage approach. In the first stage, the role of inflammatory markers such as monocytes, lymphocytes, CLR, CRP, neutrophils, NLR, and MLR in the classification of pulmonary toxicity was evaluated using ROC analysis. The areas under the curve were found to be significant for monocytes, lymphocytes, and CLR. Optimal cut-off values were determined using Youden’s J index. The cut-off values with the highest J index were identified as the optimal cut-off values. For lymphocytes, <950; for monocytes, <130; and for CLR, >15.2 were found to favor toxicity in the classification of pulmonary toxicity. In the second model, the role of inflammatory markers with significant areas under the curve in toxicity prediction was evaluated. *p* < 0.05 value was considered statistically significant.

## 3. Results

**Figure 1:**
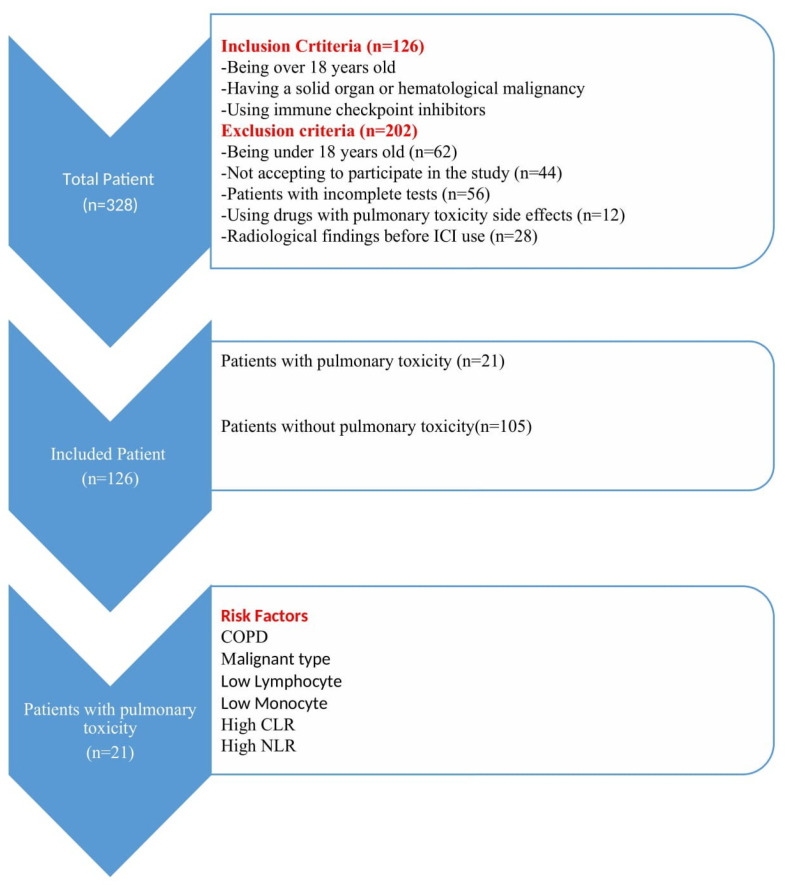
Study flow chart.

A total of 328 patients admitted to the oncology and pulmonology outpatient clinics during the relevant periods were evaluated, and 62 patients who were under 18 years of age, 44 patients who refused to participate in the study, 12 patients who were using drugs with a risk of pulmonary toxicity, 28 patients with pathologic radiologic findings before treatment, and 56 patients with incomplete examinations were excluded from the study. A total of 126 patients were included in the study. The selection of participants, clinical and laboratory findings, and study model indicating the patients with pulmonary toxicity are presented in Figure 1.

The mean age of the patients included in the study was 62.93 ± 12.94 years, and 81% were male. In our study, pulmonary toxicity was detected in 21 (16.7%) of 126 patients treated with ICI. At the end of the two-year follow-up period, the all-cause mortality rate was 68.3%, and 16 (76.2%) of the 21 patients with pulmonary toxicity died during this follow-up period. The most common malignancies among the patients included in our study were lung cancer (41.3%) and malignant melanoma (28.6%). Approximately 19% of patients received ICIs for less than 6 months, while the rest received ICIs for more than 6 months. The kind of cancer (especially lung cancer), COPD, and long-term ICI use have all been found to be significantly associated with pulmonary toxicity in preliminary investigations. Respiratory and systemic symptoms of the patients, especially dyspnea, cough, and sputum, were found to be associated with pulmonary toxicity, and no significant relationship was found between pulmonary function tests and pulmonary toxicity. The impacts of sociodemographic data, symptoms, and respiratory functions on pulmonary toxicity are shown in Table 1.

Detection of nodule mass (on thoracic CT, round opacity/density increases below 30 mm were defined as nodules and those above 30 mm were defined as masses) in radiological imaging before ICI was found to be significant in terms of pulmonary toxicity. The most common radiological patterns related to ICI were organizing pneumonia (47%) and interstitial pneumonia patterns (23.8%). The relationship between radiological findings before and after immunotherapy and pulmonary toxicity is presented in Table 2. Among the laboratory parameters, we found that low lymphocytes, low monocytes, high CRP levels, and a high CRP-to-lymphocyte ratio (CLR) were associated with lung toxicity (*p* = 0.023; *p* = 0.024; *p* = 0.036; and *p* = 0.011, respectively). The relationship between laboratory parameters and pulmonary toxicity is presented in Table 2.

The model created in the logistic regression analysis, which was developed to predict the risk of pulmonary toxicity, was found to be significant (omnibus test *p* < 0.001). The dependent variable of the model is the presence of pulmonary toxicity. The independent variables are lymphocyte count, monocyte count, lymphocyte–monocyte ratio (LMR), neutrophil–lymphocyte ratio (NLR), CRP–lymphocyte ratio (CLR), CRP, presence of nodules or masses, symptoms such as cough, sputum, and dyspnea, duration of immune checkpoint inhibitor use, COPD, smoking, and lung cancer. Among the variables included in the model, NLR, CLR, cough, and the presence of COPD made significant contributions. A one-unit increase in NLR reduced the risk of toxicity by 1.064 times (OR: 0.939), a one-unit increase in CLR increased it by 1.002 times, the presence of cough symptoms increased it by 7.70 times, and the presence of COPD increased it by 4.57 times. The regression analysis performed is shown in Table 3.

**Figure 2:**
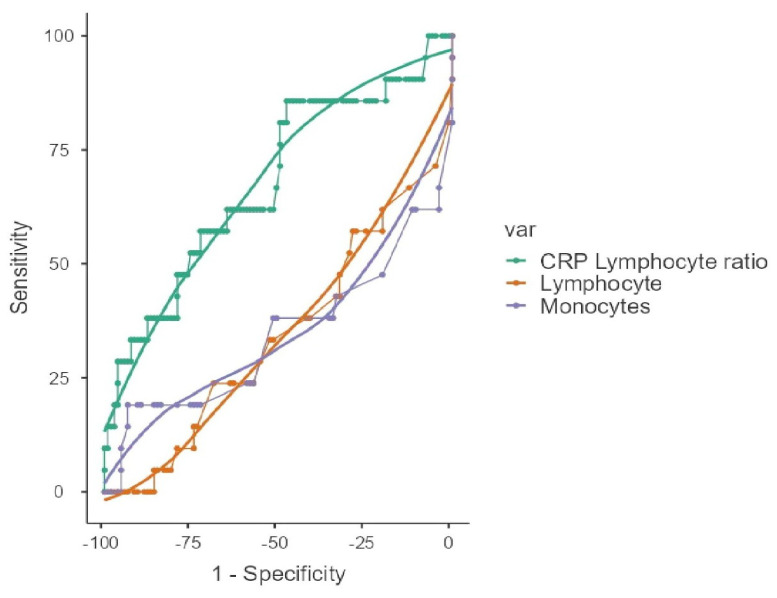
Areas under the curve (AUC) of lymphocytes, monocytes, and CRP–lymphocyte ratio.

An ROC analysis was performed to evaluate the diagnostic roles of lymphocyte, monocyte, and CLR values in pulmonary toxicity classification. According to the results of the ROC analysis, it was found that the areas under the curve for three parameters were significant, and their power as diagnostic tests in pulmonary toxicity classification was low (AUC: 0.677, 0.655, and 0.657, respectively). According to the results of our study, the optimum cut-off values were found to be 15.2 for CLR (values above the cut-off value are diagnostic for pulmonary toxicity), 130 for monocytes (values below the cut-off value are diagnostic for pulmonary toxicity), and 950 for lymphocytes (values below the cut-off value are diagnostic for pulmonary toxicity) (Table 4 and Figure 2). When the areas under the curve were compared according to the Delong test results, no diagnostic superiority was found between the tests (*p*-value = 0.897).

The logistic regression model to predict the risk of pulmonary toxicity was found to be significant (omnibus test *p* > 0.001). The model’s performance was evaluated by ROC analysis, and AUC = 0.730, indicating that the performance of the model was adequate for pulmonary toxicity classification. The dependent variable of the model was the pulmonary toxicity status, and the independent variables were lymphocytes (risk group < 950), monocytes (risk group < 130), and CLR (risk group > 15.2), which were created according to the cut-off values found in our study. Among the variables included in the model, the monocyte value made a significant contribution to the model, and the risk of pulmonary toxicity was found to be 11.75 times higher in patients with a hospital admission monocyte value <130 (Table 4).

## 4. Discussion

The present study showed that the pulmonary toxicity rate due to ICIs use was 16.7%, and the all-cause mortality rate was approximately 68.3% at the end of the 2-year follow-up. The type of malignancy (especially lung cancer); COPD; long-term ICI use; symptoms such as dyspnea, cough, and sputum; radiological detection of nodules or masses in the lung before ICI use; and laboratory parameters such as a low lymphocyte count, a low monocyte count, high CRP levels, and high a CLR were found to be associated with pulmonary toxicity. With the risk analysis, cough, COPD, NLR, and CLR were determined as risk factors for ICI-related pulmonary toxicity. We found that the risk of pulmonary toxicity increased at cut-off values of 15.2 and above for CLR, 130 and below for monocytes, and 950 and below for lymphocytes. The risk of pulmonary toxicity was 11.75 times higher, especially when the monocyte value was below 130.

Although there is different information in the literature regarding the incidence of pulmonary toxicity due to ICIs, incidence rates between 1% and 12% are mentioned. A recent study of 1004 patients between January 2014 and February 2023 found that the risk of ICI-related pneumonitis was 2% and the most common radiological pattern was organizing pneumonia [11]. According to a meta-analysis, the overall incidence of ICI-related interstitial lung disease (ICI-ILD) was 2.7% for all grades and 0.8% for the most severe grades (grade ≥ 3). In the same study, the overall incidence of ICI-ILD for all grades was between 1.4% and 5.8% in NSCLC studies, between 1% and 4.4% in melanoma studies, and between 2.7% and 4.8% in renal cell carcinoma studies [12]. In three different studies, the incidence rates of ICI-related ILD were found to be 3.5%, 5%, and 11.8%, respectively, indicating a clear heterogeneity in this regard [13,14,15]. The present study found the rate of pulmonary toxicity due to ICI to be 16.7%. This result was higher than the data in the literature, and it was thought that genetic factors as well as regional and geographical differences could be effective in this situation. The presence of certain comorbidities (especially rheumatologic diseases, autoimmune diseases, or certain chronic inflammatory diseases) or previous treatments that have not been proven to cause pulmonary toxicity or unspecified medical treatments may also make this patient group more susceptible to toxicity and should be considered and reviewed. Additionally, it should not be ignored that the patients included in this study were patients who needed treatment in a tertiary health institution.

As observed in melanoma studies, it is well known that the incidence is increased in patients treated with combination immunotherapy. The incidence of pneumonitis was higher in combination regimens (including nivolumab and ipilimumab given simultaneously or sequentially or nivolumab plus peptide vaccines) compared to monotherapy (6.6%) [12]. Since the incidence is also higher in combined therapies than in monotherapy, patients treated with bi-therapy (anti-PD-1 and CTLA-4) require close clinical and radiological follow-up [12]. However, the combination of chemotherapy and immunotherapy (pembrolizumab) has not been associated with an increased risk of pneumonitis in lung cancer [16]. Furthermore, the administration of immunotherapy (durvalumab) after chemoradiation in patients with locally advanced NSCLC was associated with an acceptable rate of lung toxicity (3.4% grade 3/4 compared with 2.6% in the placebo arm) [17,18]. In our study, we administered combination immunotherapy to a total of 16 patients, and pulmonary toxicity was observed in only 1 of them. However, we think that the small number of patients and the shorter duration of ICIs may be related to this situation.

Approximately 611,720 people are estimated to have died from cancer in the United States in 2024, with an overall mortality rate of 146.0 per 100,000 people per year [19]. Another study found that in 2012, there were an estimated 14.1 million new cancer cases and 8.2 million cancer deaths worldwide [20]. A 2018 study based on screening data from 185 countries or territories around the world found that the mortality rates for cancer patients are quite different, especially among men, with 171.0 in Eastern Europe, 67.4 in Central America, 120.7 in Melanesia, and 64.2 in East Asia (excluding China) [21]. The same study reported a 21.4% risk of developing cancer and a 17.7% risk of dying from cancer before the age of 75 for both sexes [21]. Another noteworthy point of the study is that while 5-year net survival from diagnosis is estimated to be between 10 and 20% for all types of lung cancer, the 5-year survival for patients with late-stage disease is generally estimated to be less than 5% [21]. We found the all-cause mortality rate in our patients to be 68.3% at the end of the 2-year follow-up. We believe that our mortality rates are this high because of the health policies in our country, the difficulties in accessing ICI, and the fact that the patient group in which we used immunotherapy treatment consisted of intermediate–advanced-stage patients.

Studies have noted that patients present with a wide range of symptoms, including dyspnea, non-productive cough, tachypnea, fever, and malaise, while more severe cases may present with progressive dyspnea with severe hypoxemia and respiratory failure [22,23,24]. In our study, symptoms such as shortness of breath, cough, and sputum production were observed to be associated with pulmonary toxicity, and these findings are consistent with the literature. We found that the risk of ICI-related pulmonary toxicity may increase by 7.7 times, especially in the presence of cough symptoms, and that cough is ultimately the most important symptom of pulmonary toxicity.

Some studies have been conducted at different times for risk factors for pulmonary toxicity due to ICI. In a Phase I study by Topalian et al. [25] to evaluate the safety, antitumor activity, and pharmacokinetics of nivolumab, they enrolled 296 patients and found no clear relationship between the occurrence of pneumonitis and tumor type, drug dose level, or number of doses received [25]. A review reported that chemotherapy-induced lung inflammation, prior radiotherapy, pre-existing lung disease, and smoking may be risk factors contributing to the occurrence, severity, and prognosis of pulmonary toxicity [26]. A study in patients with lung cancer noted that exposure to tobacco smoke or chronic lung diseases may limit pulmonary resistance to external stressors, thereby increasing pulmonary toxicity [18]. Another study indicated that thoracic radiotherapy may potentiate lung toxicity by increasing local inflammation and releasing tumor antigens into lung tissues [27]. This study found that the malignancy type (lung cancer) and underlying chronic lung disease (COPD) are risk factors for ICI-related pulmonary toxicity. Our risk analyses revealed that the presence of COPD, in particular, causes a 4.7-fold increase in the risk of pulmonary toxicity. Our current findings are consistent with the literature, and we must not forget that comorbidities, particularly the presence of COPD, are important in the development of ICI-related pulmonary toxicity.

There are many studies examining the association of some biomarkers in blood with irAEs. Egami et al. [28] reported that 2 weeks after the start of ICI treatment, an increase in absolute lymphocyte count was significantly associated with an increased risk of irAEs, while Matsukane et al. [29] reported that an increased NLR can predict the severity of subsequent irAE pneumonia with high accuracy, and the higher the NLR value is, the more severe irAEs will occur. In another study, it was determined that an LMR > 1.6 before treatment significantly reduced the risk of irAE, and an NLR > 2.3 was significantly associated with an increased risk of irAE [30]. Another study noted that the incidence of irAEs was significantly associated with a higher baseline absolute monocyte count [31]. According to our study, although there are partial increases in risk with a CLR increase and NLR decrease, low lymphocyte and monocyte counts in particular have been identified as serious risk factors for pulmonary toxicity. Monocyte counts below 130 and lymphocyte counts below 950 are associated with a significant increase in risk. During follow-up of these patients in the stable phase, if lymphocyte and monocyte counts fall below the specified values, they should be evaluated more carefully for pulmonary toxicity, which is supported by clinical findings. For this purpose, we recommend that even mild or initial radiological findings supporting pulmonary toxicity accompanying lymphocyte and monocyte counts below the cut-off values be taken into account and that ICI treatment be modified or discontinued based on these findings. In our study, we found no evidence in the literature that the cut-off values we established for lymphocyte and monocyte counts increase the risk of pulmonary toxicity or irAE.

The limitations of our study include the fact that it was conducted in a single center, most of the patients included in the study were patients with intermediate and advanced cancer, the sample size was small, and the follow-up period was short. Therefore, multicenter studies with larger participation and longer periods are needed.

## 5. Conclusions

With the increasing use of ICIs today, it is inevitable that the incidence of pulmonary toxicity will increase, as will other side effects. In our study, we found that pulmonary toxicity due to ICI use was more frequent than expected and that COPD, lung cancer, low lymphocyte and low monocyte counts, and a high CLR were risk factors for pulmonary toxicity due to ICI use. Among these risk factors, the most striking result we found in our study was that the risk of pulmonary toxicity increased 11.75-fold in patients with a monocyte count of 130 and below. These findings underscore the importance of incorporating routine hematologic and clinical monitoring into the follow-up of patients receiving ICIs, especially those with predisposing risk factors. Given the potential for late recognition of pulmonary toxicity to increase morbidity and mortality, clinicians should adopt a proactive approach: patients with suspicious clinical or laboratory features should undergo early diagnostic evaluation, including imaging and, if necessary, bronchoscopy. Treatment modification or discontinuation may be warranted in such cases to prevent severe complications. Early identification and intervention may allow for more effective management of ICI-related pulmonary toxicity and ultimately reduce mortality. Our findings support the integration of risk-based monitoring strategies into clinical practice for patients undergoing immunotherapy.

## Figures and Tables

**Table 1:** Baseline sociodemographic and clinical characteristics in relation to pulmonary toxicity.

Variables		*n* (%) or Mean ± SD			*p*
		Total (*n* = 126)	Toxicity (+) (*n* = 21)	Toxicity (−) (*n* = 105)	
Age		62.93 ± 12.94	63.10 ± 14.24	62.90 ± 12.73	0.532
Sex	Male	102 (81)	20 (95.2)	82 (78.1)	0.068
	Female	24 (19)	1 (4.8)	23 (21.9)	
Smoke	Yes	86 (68.3)	18 (85.7)	68 (64.8)	0.060
	No	40 (31.7)	3 (14.3)	37 (35.2)	
Type of Malignancy	Lung Cancer	52 (41.3)	13 (61.9)	39 (37.1)	0.000
	Malignant Melanoma	36 (28.6)	1 (4.8)	35 (33.3)	
	Kidney and Bladder	11 (8.7)	1 (4.8)	10 (9.5)	
	Lymphoma	17 (13.5)	1 (4.8)	16 (15.2)	
	Mesothelioma	4 (3.2)	3 (14.3)	1 (1)	
	Nasopharynx	2 (1.6)	0 (0)	2 (1.9)	
	Esophagus	1 (0.8)	1 (4.8)	0 (0)	
	Testis	1 (0.8)	0 (0)	1 (1)	
	Prostate	1 (0.8)	1 (4.8)	0 (0)	
	Hepatocellular Carcinoma	1 (0.8)	0 (0)	1 (1)	
COPD	Yes	41 (32.5)	11 (52.4)	30 (28.6)	0.034
	No	85 (67.5)	10 (47.6)	75 (71.4)	
HT	Yes	40 (31.7)	6 (28.6)	34 (32.4)	0.732
	No	86 (68.3)	15 (71.4)	71 (67.6)	
DM	Yes	34 (27)	6 (28.6)	28 (26.7)	0.858
	No	92 (73)	15 (71.4)	77 (73.3)	
Heart Diseases	Yes	32 (25.4)	5 (23.8)	27 (25.7)	0.855
	No	94 (74.6)	16 (76.2)	78 (74.3)	
Chronic Liver Disease	Yes	11 (8.7)	1 (4.8)	10 (9.5)	0.398
	No	115 (91.3)	20 (95.2)	95 (90.5)	
Rheumatological Diseases	Yes	7 (5.6)	3 (14.3)	4 (3.8)	0.056
	No	119 (94.4)	18 (85.7)	101 (96.2)	
Chronic Kidney Disease	Yes	3 (2.4)	0 (0)	3 (2.9)	0.083
	No	123 (97.6)	21 (100)	102 (97.1)	
Neurological Diseases	Yes	7 (5.6)	1 (4.8)	6 (5.7)	0.862
	No	119 (94.4)	20 (95.2)	99 (94.3)	
CCI Risk Score	Low	22 (17.5)	2 (9.5)	20 (19)	0.589
	Medium	15 (11.9)	3 (14.3)	12 (11.4)	
	High	42 (33.3)	6 (28.6)	36 (34.3)	
	Very High	47 (37.3)	10 (47.6)	37 (35.2)	
RT	Yes	16 (12.7)	4 (19)	12 (11.4)	0.425
	No	110 (87.3)	17 (81)	93 (88.6)	
Indication for ICI	Early-Stage Malignancies	43 (34.1)	10 (47.6)	33 (31.4)	0.351
	Advanced-Stage Malignancies	50 (39.7)	7 (33.3)	43 (41)	
	Relapse	33 (26.2)	4 (19)	29 (27.6)	
ICI usage time	<3 months	2 (1.6)	2 (9.5)	0 (0)	0.010
	3 months–6 months	22 (17.5)	5 (23.8)	17 (16.2)	
	6 months–1 year	56 (44.4)	7 (33.3)	49 (46.7)	
	>1 year	46 (36.5)	7 (33.3)	39 (37.1)	
Time to ICI-Related Radiological Abnormalities	No	91 (72.2)	0 (0)	91 (86.7)	0.000
	0–6 months	7 (5.6)	2 (9.5)	5 (4.8)	
	6 months–1 year	13 (10.3)	7 (33.3)	6 (5.7)	
	>1 year	15 (11.9)	12 (57.1)	3 (2.9)	
Second ICI (Durvalumab, İpilimumab) Use	Yes	16 (12.7)	1 (4.8)	15 (14.3)	0.231
	No	110 (87.3)	20 (95.2)	90 (85.7)	
Fever	Yes	11 (8.7)	2 (9.5)	9 (8.6)	0.888
	No	115 (91.3)	19 (90.5)	96 (91.4)	
Weakness–Fatigue	Yes	74 (58.7)	14 (66.7)	60 (57.1)	0.412
	No	52 (41.3)	7 (33.3)	45 (42.9)	
Headache–Dizziness	Yes	49 (38.9)	9 (42.9)	40 (38.1)	0.690
	No	77 (61.1)	12 (57.1)	65 (61.9)	
Dyspnea	Yes	68 (54)	16 (76.2)	52 (49.5)	0.025
	No	58 (46)	5 (23.8)	53 (50.5)	
Cough	Yes	65 (51.6)	17 (81)	48 (45.7)	0.003
	No	61 (48.4)	4 (19)	57 (54.3)	
Sputum	Yes	39 (31)	12 (57.1)	27 (25.7)	0.004
	No	87 (69)	9 (42.9)	78 (74.3)	
Chest Pain	Yes	35 (27.8)	5 (23.8)	30 (28.6)	0.657
	No	91 (72.2)	16 (76.2)	75 (71.4)	
Hemoptysis	Yes	9 (7.1)	3 (14.3)	6 (5.7)	0.164
	No	117 (92.9)	18 (85.7)	99 (94.3)	
Respiratory Function Tests	Normal	94 (74.6)	19 (90.5)	75 (71.4)	0.094
	Obstructive	17 (13.5)	2 (9.5)	15 (14.3)	
	Restrictive	15 (11.9)	0 (0)	15 (14.3)	

Abbreviations: CCI: Charlson Comorbidity Index; COPD: Chronic Obstructive Pulmonary Disease; DM: Diabetes Mellitus; HT: Hypertension; ICI: Immune Checkpoint Inhibitor; RT: Radiotherapy.

**Table 2:** Radiological and laboratory parameters associated with pulmonary toxicity.

Variables		*n* (%) or Mean ± SD			*p*
		Total (*n* = 126)	Toxicity (+) (*n* = 21)	Toxicity (−) (*n* = 105)	
Radiological Findings Before ICI Use					
Normal	Yes	72 (57.1)	17 (81)	55 (52.4)	0.016
	No	54 (42.9)	4 (19)	50 (47.6)	
Pleural Effusion	Yes	7 (5.6)	2 (9.5)	5 (4.8)	0.496
	No	119 (94.4)	19 (91.5)	100 (95.2)	
Nodule Mass	Yes	74 (58.7)	8 (38.1)	66 (62.9)	0.035
	No	52 (41.3)	13 (61.9)	39 (37.1)	
Cyst Cavity	Yes	9 (7.1)	3 (14.3)	6 (5.7)	0.164
	No	117 (92.9)	18 (85.7)	99 (94.3)	
Radiological findings after ICI use					
OP	Yes	12 (9.5)	12 (57.1)	0 (0)	0.000
	No	114 (90.5)	9 (42.9)	105 (100)	
IIP/NSIP	Yes	5 (4)	5 (23.8)	0 (0)	0.000
	No	121 (96)	16 (76.2)	105 (100)	
HP	Yes	3 (2.4)	3 (14.3)	0 (0)	0.000
	No	123 (97.6)	18 (85.7)	105 (100)	
Wbc (×10^3^/mm^3^)		10.32 ± 7.84	8.42 ± 6.99	10.69 ± 7.97	0.229
Hemoglobin		11.61 ± 4.09	11.25 ± 2.82	11.67 ± 4.30	0.183
Hematocrit		34.30 ± 7.43	33.88 ± 8.51	34.38 ± 7.24	0.416
Platelet (×10^3^/mm^3^)		254.30 ± 144.25	220.57 ± 137.79	261.05 ± 145.20	0.361
Neutrophil		8090.40 ± 7448.90	6849.52 ± 6381.49	8338.57 ± 7647.58	0.176
Lymphocyte		1272.34 ± 973.93	850.71 ± 651.02	1356.67 ± 1007.72	0.023
Monocyte		721.94 ±551.64	554.05 ± 536.27	755.52 ± 551.01	0.024
Eosinophil		162.62 ± 234.35	119.52 ±124.27	171.24 ±250.18	0.418
LMR		2.30 ±1.99	2.95 ± 3.36	2.17 ± 1.57	0.503
NLR		11.67 ± 15.29	13.56 ± 18.01	11.29 ± 14.75	0.369
CLR		294.63 ±1815.92	1273.81 ± 4380.35	98.79 ±202.93	0.011
CRP		70.55 ±97.35	110.63 ±121.72	62.53 ±90.28	0.036
Ferritin		646.33 ± 1671.64	795.10 ±1758.66	616.58 ± 1660.86	0.487

Abbreviations: ICIs: immune checkpoint inhibitors; HP: hypersensitivity pneumonitis; IIP: idiopathic interstitial pneumonia pattern; NSIP: idiopathic non-specific interstitial pneumonia; OP: organized pneumonia; Wbc: White blood cell; LMR: lymphocyte–monocyte ratio; NLR: neutrophil–lymphocyte ratio; CLR: C-reactive protein–lymphocyte ratio; CRP: C-reactive protein.

**Table 3:** Regression analysis of risk factors for ICI-associated pulmonary toxicity.

R^2^ = 0.353	B	*p*	O.R.	95% C.I. for OR.	
				Lower	Upper
NLR	−0.063	0.031	0.939	0.887	0.994
CLR	0.002	0.007	1.002	1.001	1.004
Cough (ref:no)	2.042	0.004	7.705	1.897	31.288
COPD (ref:no)	1.520	0.010	4.571	1.436	14.552
Constant	−1.657	0.000	0.191		

Abbreviations: CLR: C-reactive protein–lymphocyte ratio; COPD: chronic obstructive pulmonary disease; ICI: immune checkpoint inhibitor; NLR: neutrophil-lymphocyte ratio.

**Table 4:** ROC analysis and univariate logistic regression analysis of lymphocyte count, monocyte count, and CRP–lymphocyte ratio in pulmonary toxicity evaluation.

ROC Analysis								
Scale		Cut-Off Point	Sensitivity (%)	Specificity (%)	PPV (%)	NPV (%)	Youden’s Index	AUC
Lymphocyte		<950	57.14	67.62	26.09	88.75	0.248	0.657
Monocyte		<130	38.1	96.19	66.67	88.6	0.343	0.655
CLR		>15.2	85.71	47.62	24.66	94.34	0.333	0.677
**Multivariate Logistic Regression Analysis**								
					95% Confidence Interval			
Predictor	B	SE	*p*	Odds ratio	Lower	Upper	VIF	Tolerance
Intercept	−2.488	0.522	<0.001	0.0830	0.0299	0.231		
Lymphocyte	−0.488	0.675	0.470	0.6142	0.1637	2.304	1.61	0.622
Monocytes	2.464	0.825	0.003	11.7569	2.3344	59.211	1.43	0.700
CLR	1.071	0.664	0.107	2.9183	0.7942	10.724	1.22	0.822

Abbreviations: CLR: C-Reactive Protein–Lymphocyte Ratio.

## Data Availability

The data presented in this study are available upon request from the corresponding author due to confidentiality restrictions.
